# Mycoplasma genitalium Protein of Adhesion Suppresses T Cell Activation via CypA-CaN-NFAT Pathway

**DOI:** 10.1128/spectrum.04503-22

**Published:** 2023-04-19

**Authors:** Dan Luo, Haodang Luo, Xiaoliang Yan, Aihua Lei, Jun He, Yating Liao, Kailan Peng, Xia Li, Youyuan Ye, Li Chen, Zhuo Zeng, Hua Xiao, Yanhua Zeng

**Affiliations:** a Institute of Pathogenic Biology, Basic Medical School, Hengyang Medical School, University of South China, Hengyang, Hunan, China; b Hunan Provincial Key Laboratory for Special Pathogens Prevention and Control, Hengyang, Hunan, China; c Hunan Province Cooperative Innovation Center for Molecular Target New Drug Study, Hengyang, Hunan, China; d Department of Clinical Laboratory, the Affiliated Nanhua Hospital, Hengyang Medical School, University of South China, Hengyang, Hunan, China; Wayne State University

**Keywords:** CaN-NFAT signaling, *M. genitalium* protein of adhesion, T cell activation, cyclophilin A

## Abstract

Mycoplasma genitalium is a prokaryotic microorganism that causes urogenital tract infections. M. genitalium protein of adhesion (MgPa) was essential for M. genitalium attachment and subsequent invasion into host cells. Our prior research confirmed that Cyclophilin A (CypA) was the binding receptor for MgPa and MgPa-CypA interaction can lead to the production of inflammatory cytokines. In this study, we revealed that the recombinant MgPa (rMgPa) could inhibit the CaN-NFAT signaling pathway to reduce the level of IFN-γ, IL-2, CD25, and CD69 in Jurkat cells by binding to the CypA receptor. Moreover, rMgPa inhibited the expressions of IFN-γ, IL-2, CD25, and CD69 in primary mouse T cells. Likewise, the expressions of these T cells activation-related molecules in CypA-siRNA-transfected cells and CypA^−/−^ mouse primary T cell was strengthened by rMgPa. These findings showed that rMgPa suppressed T cell activation by downregulating the CypA-CaN-NFAT pathway, and as a result, acted as an immunosuppressive agent.

**IMPORTANCE** Mycoplasma genitalium is a sexually transmitted bacterium that can co-infect with other infections and causes nongonococcal urethritis in males, cervicitis, pelvic inflammatory disease, premature birth, and ectopic pregnancy in women. The adhesion protein of M. genitalium (MgPa) is the primary virulence factor in the complicated pathogenicity of M. genitalium. This research proved that MgPa could interact with host cell Cyclophilin A (CypA) and prevent T cell activation by inhibiting Calcineurin (CaN) phosphorylation and NFAT nuclear translocation, which clarified the immunosuppression mechanism of M. genitalium to host T cells. Therefore, this study can provide a new idea that CypA can be used for a therapeutic or prophylactic target for M. genitalium infection.

## INTRODUCTION

Mycoplasma genitalium, a prokaryotic microorganism that causes urogenital tract infections, can cause cervicitis, pelvic inflammatory illness in women, and urethritis in men, as well as increase the risk of transmission of the human immunodeficiency virus (HIV), Chlamydia trachomatis, and *Neisseria gonorrhea* (1–6). Studies demonstrated that M. genitalium could induce persistent infection in human endocervical epithelial cells and thus lead to the chronic inflammatory response (7). Long-term survival of M. genitalium in the host may be correlated with its capacity to escape or even compromise the immune system. However, the molecular mechanisms establishing persistent M. genitalium infection and its immunosuppressive agent remain unknown.

M. genitalium is an extracellular parasite, as was previously documented. However, a prior study established that M. genitalium could survive for 7 days and proliferate intracellularly in mammalian cells (8). Ueno PM et al. demonstrated M. genitalium could localize around the nucleus and enter the nucleus after infecting host cells for 30 min (9). The researchers also learned that M. bovis was present in T and B cells, and that it inhibited the response of bovine lymphocytes (10). Above studies indicated that some mycoplasmas could invade host cells and evade immune cell clearance to cause persistent infection. For instance, M. genitalium can avoid phagocytosis by suppressing neutrophils' respiratory bursts, decreasing the generation of nitric oxide and reactive oxygen species, destroying extracellular bactericidal networks, and encouraging neutrophil reproduction (11, 12). In addition, M. genitalium could inhibit the activation of lymphocytes by inducing host cells to express the anti-inflammatory cytokine Interleukin-10, downregulating the activity of host cell nuclear transcription factors, and regulating adverse co-stimulatory signals in immune cells (13). However, whether M. genitalium can directly playing immunosuppressive role to T cell remains unclear. Therefore, we focused on the immunosuppressive effect of M. genitalium on T cells activation and its potential mode of action.

The most crucial protein for the pathogenesis of M. genitalium in terms of adherence and colonization is the adhesion protein (MgPa) of the pathogen (14). Our prior research verified that cyclophilin A (CypA) on human urethral epithelial cells membrane is the primary binding receptor for MgPa (15). CypA, the major member of cyclophilin family, is mainly distributed in the membrane and intracellular space of prokaryotic and eukaryotic cells. Moreover, CypA can specifically bind to Cyclosporin A (CsA) to form a CypA-CsA compound and thus inhibits T cells activation (16). However, whether MgPa-CypA interaction can also inhibit T cell activation through a similar mechanism as CypA-CsA compound remains unclear. Studies have shown that T cells can be activated via CypA-Calcineurin (CaN)-nuclear factor of activated T cells (NFAT) signaling pathway. CaN is serine/threonine protein phosphatase regulated by calmodulin. Interferon-gamma (IFN-γ) and interleukin-2 (IL-2) gene transcription can be induced by CaN activation through the dephosphorylation of the nuclear factor of activated T cells (NFAT) (17). Other studies have confirmed that CaN is a therapeutic target, and its inhibitors, such as CsA and FK506 have been used as immunosuppressants (18). Since IL-2 and INF-γ are the regulators of T cells' activation and proliferation, we hypothesized that the MgPa-CypA connection could affect the production of these cytokines by blocking the CaN-NFAT signaling pathway, hence inhibiting T cell activation.

Overall, our findings suggested that MgPa suppresses T cell activation through the CypA-CaN-NFAT signaling pathway, which further elucidates the biological functions of MgPa and the possible molecular immunosuppressive mechanism of M. genitalium.

## RESULTS

### The distribution of CypA and co-location between of CypA and rMgPa in Jurkat cells.

**FIG 1:**
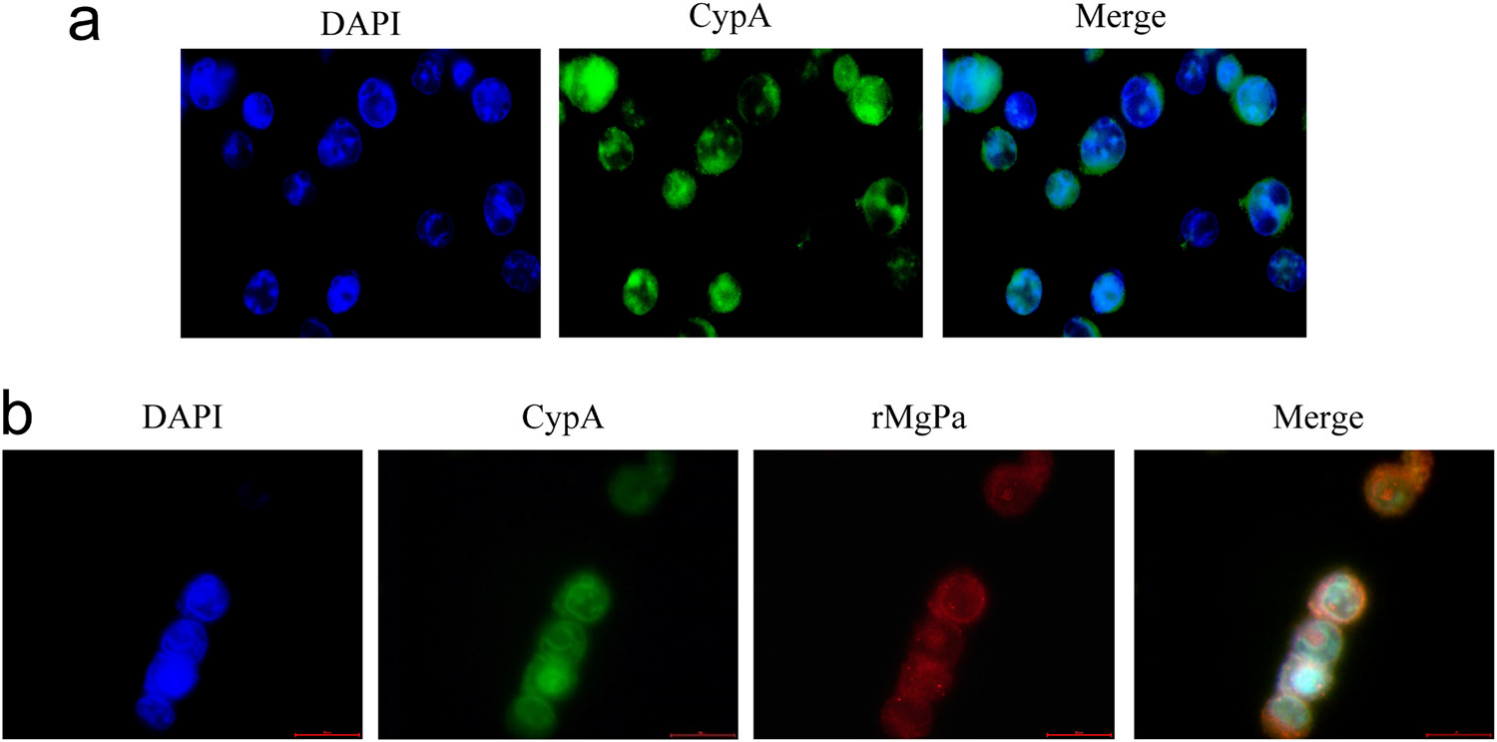
Co-location of CypA and rMgPa in Jurkat cells by IFA. Jurkat cells were cultured on coverslips in a 24-well plate with anti-CypA and FITC-conjugated goat anti-mouse IgG(H+L) antibodies. Successively the cells were dyed with DAPI (panel a). For co-location assay, the cells were stimulated with 30 μg/mL of rMgPa, and then incubated with anti-rMgPa and anti-CypA antibodies, followed by incubation with cy3-conjugated goat anti-rabbit antibody and FITC-conjugated AffiniPure goat anti-mouse IgG (H+G) (panel b). All results were observed using a fluorescence microscope (×1000).

It has been demonstrated that CypA is widely expressed in various cells. IFA was used to identify the distribution of CypA in Jurkat cells in order to determine whether CypA is also present in Jurkat cells. The results are shown in Fig. 1a, where green fluorescence was observed in the cell membrane and cytoplasm, and the nucleus was stained into blue, which demonstrated the CypA distribute in the cell membrane and cytoplasm.

Then, the co-localization between of rMgPa and CypA was examined using double immunofluorescence staining. When observed using a fluorescence microscope, rMgPa protein showed a red fluorescent signal, and CypA displayed a green fluorescent, and the nucleus was dyed blue by DAPI. In Fig. 1b, it is shown that rMgPa and CypA were mainly distributed on the cell membrane and partly distributed in the cytoplasm and nucleus. After being synthesized by the image software, red and yellow signals were observed, demonstrating the co-location between of rMgPa and CypA in the Jurkat cells.

### rMgPa inhibited the CaN phosphorylation in Jurkat cells.

**FIG 2:**
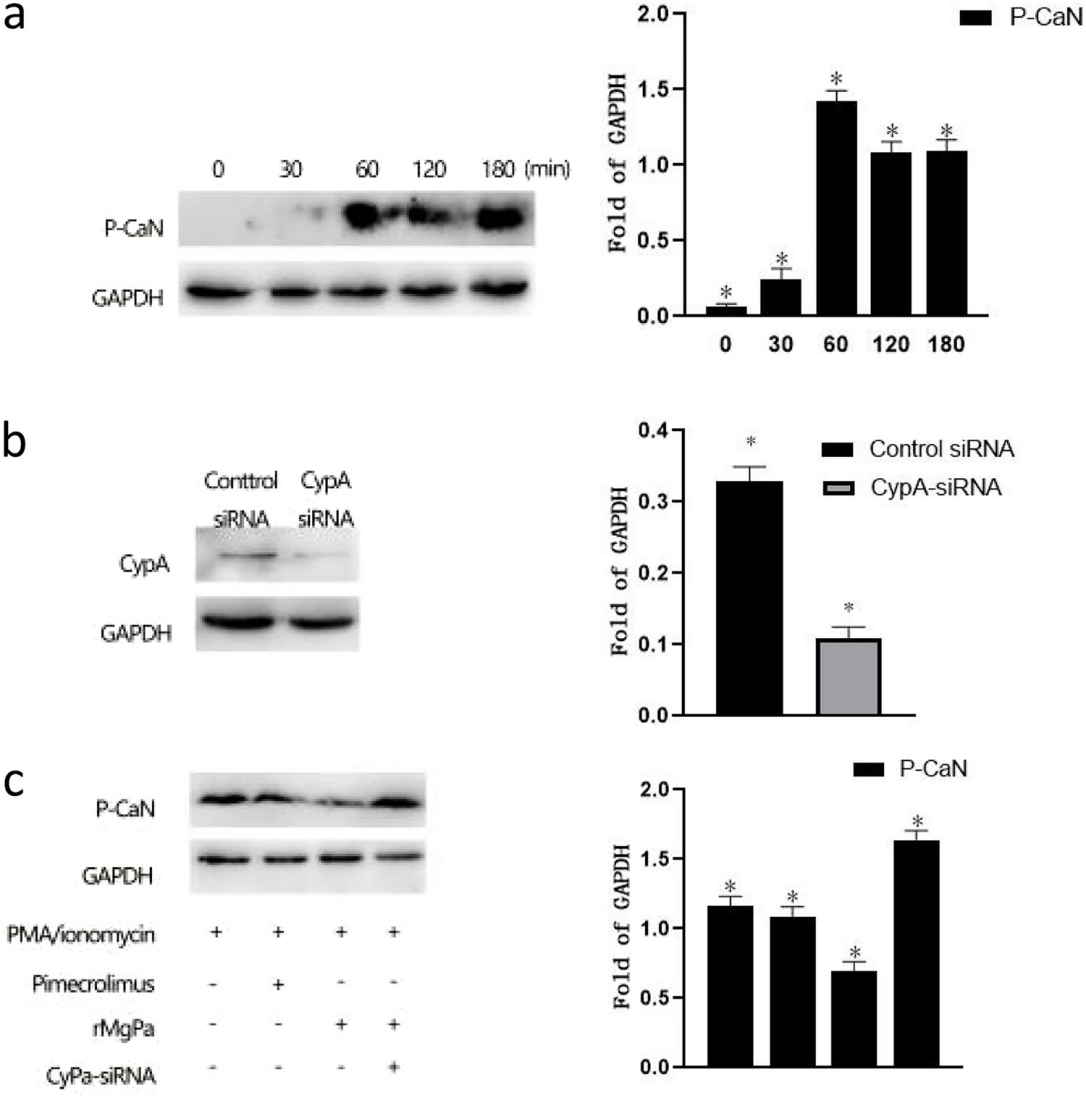
rMgPa affect the CaN in Jurkat cells. Jurkat cells were stimulated with PMA/ionomycin at different times (0, 30, 60, 120, and 180 min), and the expression of CaN phosphorylation was detected by Western blotting (panel a). Jurkat cells were transfected with CypA or scrambled siRNA and the interference efficiency was detected by Western blotting (panel b). The Jurkat cells were pretreated with PMA/ionomycin, Pimecrolimus, rMgPa, CypA-siRNA, respectively, and then stimulated with PMA/ionomycin in order to detect the CaN phosphorylation was measured using Western blot (panel c). The gray value of each band were analyzed using ImageJ software. *, *P* < 0.05 denotes significant differences compared with the negative group.

To determine the level of CaN phosphorylation after Jurkat cells were activated at different times, the Jurkat cells were pretreated with PMA/ionomycin to induce CaN phosphorylation, and subsequently the expression of CaN phosphorylation was detected by Western blotting. According to the results, CaN phosphorylation level started at 30 min, and its greatest expression level was at 60 min (Fig. 2a).

Western blotting was then used to measure the expression of CaN phosphorylation in Jurkat cells in order to confirm the impact of rMgPa-CypA interaction on CaN phosphorylation in Jurkat cells. As shown in Fig. 2b, the band of CypA-siRNA transfection group was significantly weaker than that of the control siRNA group, indicating that CypA-siRNA could better interfere with the expression of intracellular CypA. According to the result of Fig. 2c, compared with the PMA/ionomycin activated group, the expressions of phosphorylated CaN were decreased in the Jurkat cells stimulated with PMA/ionomycin combination with pimerecorlimus or rMgPa, which demonstrated that rMgPa could inhibit the phosphorylation of CaN in Jurkat cells. Further, CypA-siRNA significantly abrogated the dephosphorylation of rMgPa, indicating that CypA played a negative regulatory role in the CaN phosphorylation. Together, these results verified that rMgPa-CypA interaction could inhibit the phosphorylation of CaN.

### rMgPa inhibited the nuclear translocation of NFAT through the CypA-CaN pathway.

**FIG 3:**
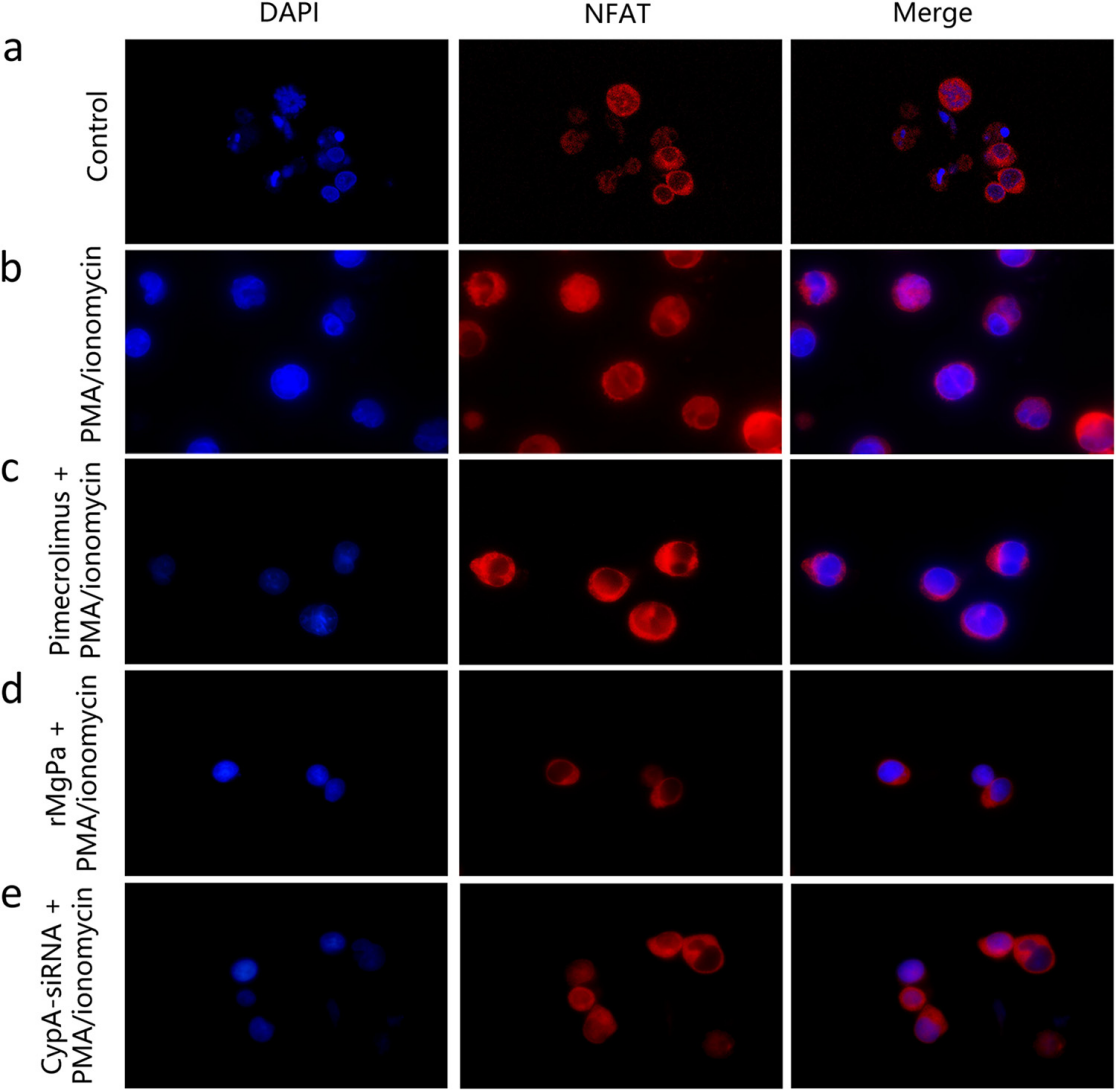
NFAT nuclear translocation in Jurkat cells was determined using IFA (×1000). The nuclear translocation of NFAT in different groups of Jurkat cells was substantiated using IFA. The NFAT distribution in control group (panel a). The NFAT was translocated into nucleus in PMA/ionomycin incubated cells (panel b). The Jurkat cells were pretreated with Pimecrolimus or rMgPa, and then the cells were stimulated with PMA/ionomycin (panels c and d). The Jurkat cells were transfected with CypA-siRNA, whereafter stimulated with PMA/ionomycin (panel e). Results were observed using a fluorescence microscope (×1000).

The effect of the rMgPa-CypA interaction on the translocation of NFATwas examined in Jurkat cells using IFA. As shown in Fig. 3b, PMA/ionomycin could promoted the translocation of NFAT from the cytoplasm into the nucleus. The nuclear translocation of NFAT was significantly suppressed by Pimecrolimus or rMgPa (Fig. 3c and d). In addition, nuclear localization of NFAT in the Jurkat cells stimulated with PMA/ionomycin combinate with rMgPa and CypA-siRNA group were enhanced (Fig. 3e). The above results suggested that the inhibition of NFAT translocation may be attributed to the rMgPa-CypA-CaN pathway.

### rMgPa modulated IL-2 and IFN-γ secretion by inhibiting CypA-CaN-NFAT in Jurkat cells.

**FIG 4:**
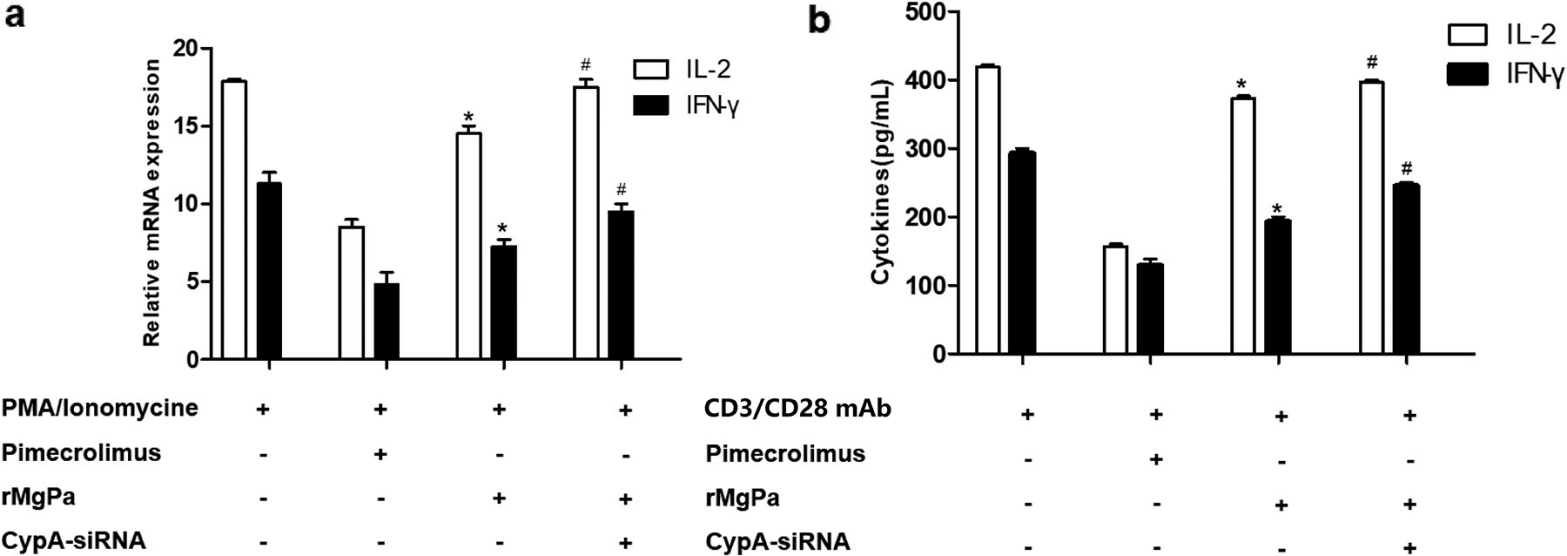
rMgPa inhibited the expression of IL-2 and IFN-γ. The Jurkat cells were pretreated with Pimecrolimus, rMgPa, or transfected with CypA-siRNA, respectively, and then stimulated with PMA/ionomycin. The mRNA levels of IL-2 and IFN-**γ** were detected using RT-qPCR (panel a). For ELISA analysis, the Jurkat cells were pretreated with Pimecrolimus, rMgPa, or transfected with CypA-siRNA, respectively, and then stimulated with CD3/CD28 MAb. The expressions of IL-2 and IFN-**γ** were detected using indirect ELISA (panel b). Compared to the group receiving rMgPa treatment, *^#^, P* < 0.05.

The previous researches supported the CaN-NFAT pathway can regulate IL-2 and IFN-γ expression. Therefore, we examined whether rMgPa could modulate IL-2 and IFN-γ secretion via CypA-CaN-NFAT pathway using RT-qPCR and indirect ELISA. As shown in Fig. 4a, compared with PMA/Ionomycin treated cells, the mRNA and protein expression of these cytokines were significantly downregulated in Jurkat cells stimulated with PMA/ionomycin combination with Pimecrolimus or rMgPa (*P < *0.05). Moreover, the mRNAs of IL-2 and IFN-γ were significantly increased in Jurkat cells stimulated with CypA-siRNA and rMgPa in the presence of PMA/ionomycin (*P < *0.05). Indirect ELISA was used to detect the secretion levels of IL-2 and IFN-γ, and the results were similar to those in Fig. 4a (Fig. 4b) (*P < *0.05). These results verified that rMgPa could inhibit IL-2 and IFN-γ production via the CypA-CaN-NFAT pathway.

### rMgPa downregulated CD69 and CD25 expression in T cells via the CypA-CaN-NFAT pathway.

**FIG 5:**
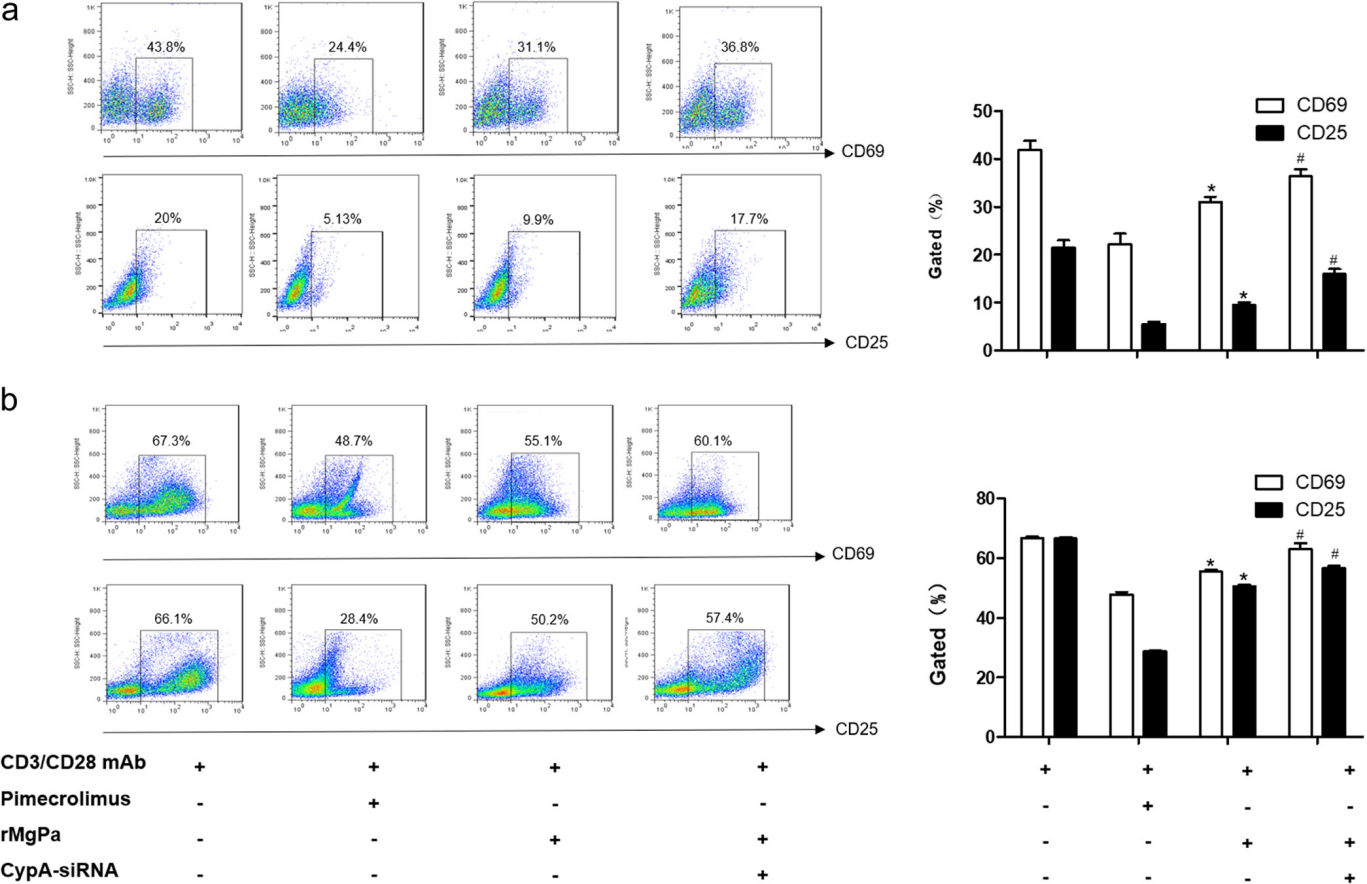
The expression of CD69 and CD25 was evaluated by FCM. Pretreatments with Pimecrolimus, rMgPa, or transfection with CypA-siRNA were applied to the Jurkat cells (panel a) and PBMCs (panel b), respectively. Then, the cells were cocultured with CD3/CD28 MAb to detect the expression of CD69 and CD25 by FCM. A *t* test was used for statistical analyses (*n* = 3). Significant differences with the CD3/CD28 MAb were designated as *, *P < *0.05. Compared to the rMgPa treated group, *^#^, P* < 0.05.

The downregulation of rMgPa-CypA interaction on CD69 and CD25 expression in Jurkat cells and PBMCs were examined using flow cytometry. As illustrated in Fig. 5a, compared with CD3/CD28 antibody treated cells, the expressions of CD69 and CD25 were significantly downregulated in Jurkat cells stimulated with CD3/CD28 antibody combinate with Pimecrolimus or rMgPa (*P < *0.05). The inhibition of T cell activation by rMgPa was partly abrogated in CypA-siRNA-transfected cells. We further tested the effect of rMgPa on the expressions of CD69 and CD25 in PBMC and obtained similar results (Fig. 5b) (*P < *0.05). These results demonstrated that the rMgPa-induced downregulation of CD69 and CD25 expressions in anti-CD3/CD28 antibody-activated T cells requires the involvement of CypA and CaN.

### rMgPa inhibited the expression of IFN-γ, IL-2, CD25, and CD69 in human primary T cells via the CypA-CaN-NFAT pathway.

**FIG 6:**
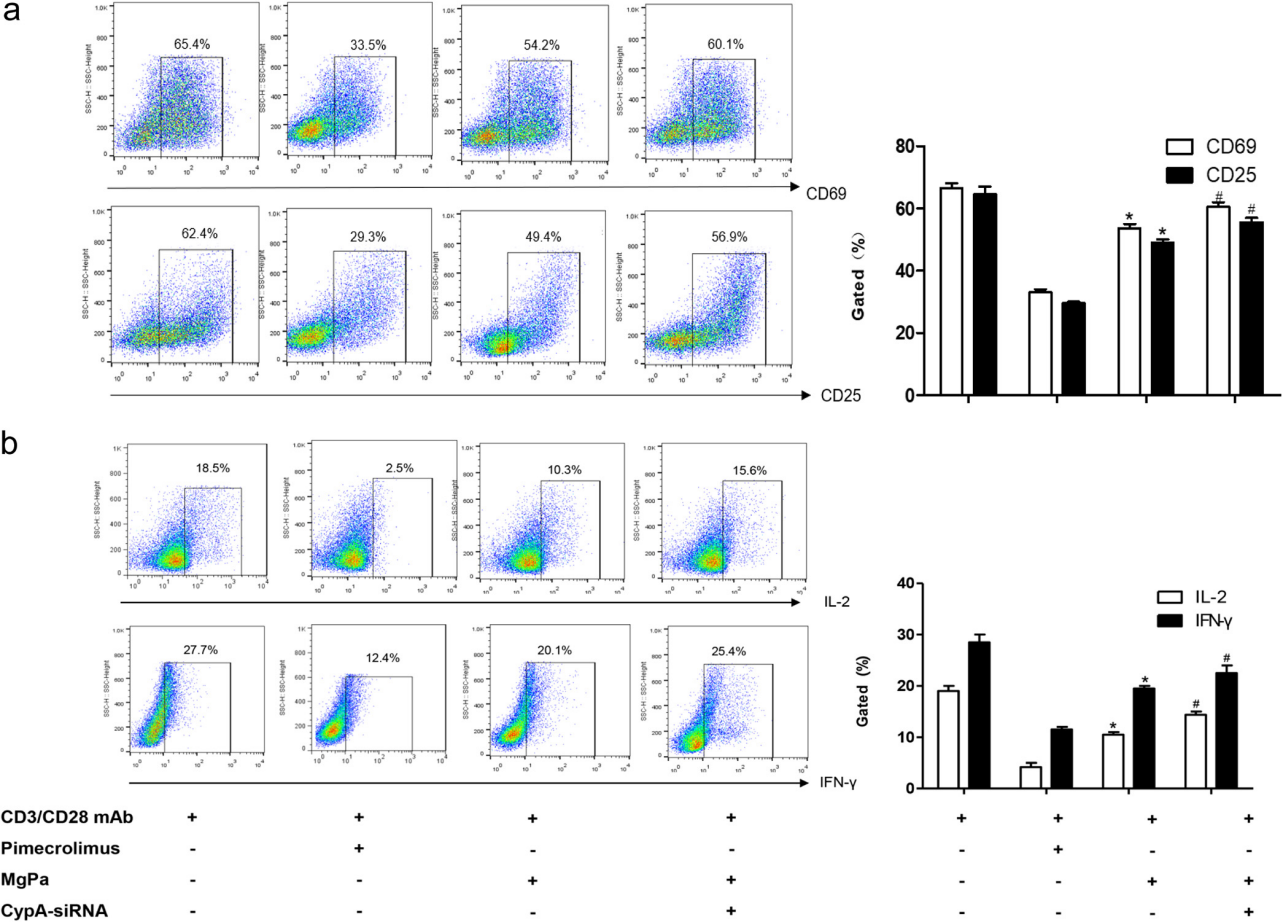
The expressions of IL-2, IFN-γ, CD69, and CD25 in human primary T cells were detected by FCM. Prior to being stimulated with CD3/CD28 MAb, the human primary T cells were pretreated with pimecrolimus, rMgPa, or transfected with CypA-siRNA, as appropriate. The expressions of CD69, CD25 (panel a) and IL-2, IFN-**γ** (panel b) were detected in human primary T cells by FCM. Significant differences with the CD3/CD28 MAb were designated as *, *P < *0.05. Compared to the rMgPa treated group, *^#^, P* < 0.05.

The inhibitory effects of the rMgPa-CypA interaction on IFN-γ, IL-2, CD25, and CD69 expressions were evaluated in purified human primary T cells using flow cytometry. Then, the mouse primary T cells was activated with CD3/CD28. As shown in Fig. 6a, for the T cells stimulated with CD3/CD28 antibody combinate with Pimecrolimus or rMgPa, the expressions of CD69 and CD25 were significantly lower than CD3/CD28 antibody treated cells (*P < *0.05). The inhibition of rMgPa on the expressions of these T cell activation-related molecules was relieved in CypA-siRNA-transfected T cells. The effect of rMgPa on the expressions of IFN-γ and IL-2 in purified human primary T cells was similar to that in Fig. 6a (Fig. 6b) (*P < *0.05). These results verified that rMgPa downregulated the IFN-γ, IL-2, CD25, and CD69 expressions in anti-CD3/CD28 activated T cells via the CypA-CaN-NFAT pathway.

### rMgPa subdued the expression of IFN-γ, IL-2, CD25, and CD69 in mouse primary T cells via CypA.

**FIG 7:**
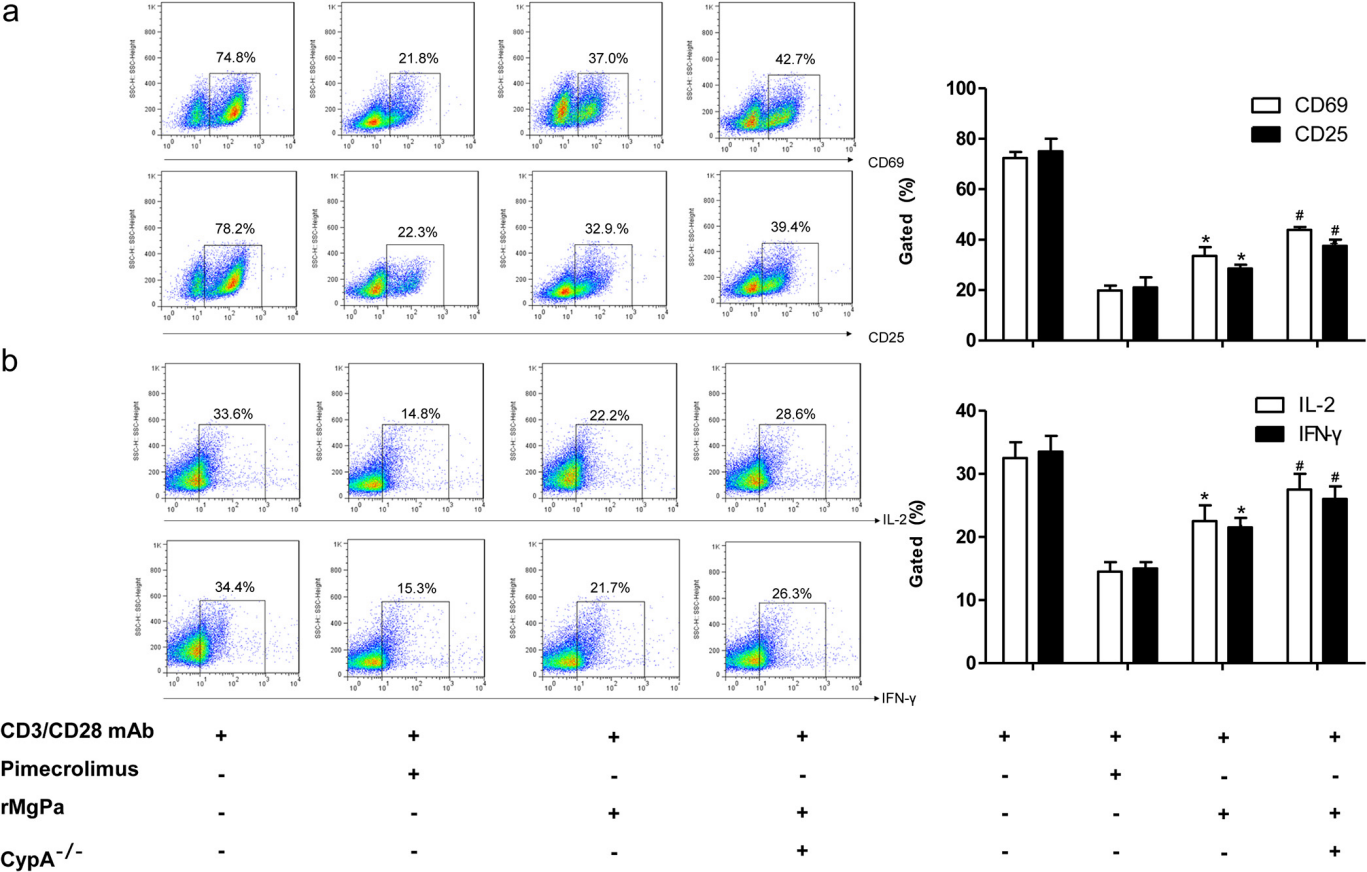
The expressions of IL-2, IFN-γ, CD69, and CD25 in mouse primary T cells were detected by FCM. The T cells were pretreated with Pimecrolimus, rMgPa, or transfected with CypA-siRNA, respectively, and then activated with CD3/CD28 MAb. The expressions of CD69, CD25 (panel a) and IL-2, IFN-**γ** (panel b) were analyzed. A *t* test was used for statistical analyses (*n* = 3). Significant differences with the CD3/CD28 MAb were designated as *, *P* < 0.05. Compared to the rMgPa treated group, ^#^, *P* < 0.05.

The amino acid homology between mouse CypA and human CypA was high, up to 95.76%. Therefore, the IFN-γ, IL-2, CD25, and CD69 expressions in normal and CypA^−/−^ mouse primary T cells were analyzed using FCM to validate whether CypA plays an essential role in the rMgPa-restrained T cell activation. As shown in Fig. 7a and b, the effect of rMgPa on the expressions of IFN-γ, IL-2, CD25, and CD69 in mouse primary T cells was similar to that of human primary T cells, which demonstrated that rMgPa reduced the production of several T cell activation-related markers in mouse T cells (*P* < 0.05). These cytokines and CD molecules were significantly elevated in CypA^−/−^ mouse primary T cells compared to rMgPa-pretreated normal mouse T cells stimulated with CD3 and CD28 antibody combinate with rMgPa (*P < *0.05). These results suggested that CypA plays an important role in the inhibition of IFN-γ, IL-2, CD25, and CD69 expressions in mouse primary T cells.

## DISCUSSION

M. genitalium is a prokaryotic microorganism that causes genitourinary system infections through sexual contact (19). The interaction between MgPa and its corresponding receptor is necessary for M. genitalium to infect host cells. Our prior research shown that CypA served as the membrane receptor for MgPa, and that the CypA-MgPa interaction could facilitate M. genitalium adhesion to human urothelial cells and cause the cells to release pro-inflammatory cytokines through the CypA-CD147-ERK-NF-κB pathway (20). We corroborated that MgPa could bind to the CypA receptor on the T cell membrane in this study, thus mediating the adhesion and invasion of M. genitalium to T cells. A previous study verified that CsA could inhibit T cell activation by binding to CypA on the T cell membrane to form the CypA-CsA complex. In the current investigation, our findings showed that MgPa could mediate the adherence of M. genitalium to human T cells and inhibit T cell activation by binding to membrane CypA and, thus, negatively regulating CaN-NFAT pathway.

HIV, Human T-Lymphoid Virus-1, Salmonella typhi*murium*, and some other pathogens could play an immunological effect on T cells in various ways. For instance, the fusion protein of gp41 and gp120 that HIV-1 uses to target various parts of the T cell receptor (TCR) complex can prevent T cells activation (21–24). Gb virus C (GBV-C) particles of Flaviviridae can inhibit T cells activation via the TCR signaling pathway mediated by the E2 protein (25). *Coxsackievirus* downregulate T cell kinase induced by interleukin-2 (IL-2) (26). Salmonella typhi*murium* L-asparaginase II inhibit T cell responses and plays a virulent role on T cells (27–29). The secretion system of *Shigella folder* type 3 can inhibit T cell migration by regulating the metabolism of host phosphocarnosin (30). Previous research established that co-incubation of Helicobacter pylori with T cells could decrease the production of IL-2 and CD25, which prevent T cells from proliferating and interrupting the cell cycle (31). M. genitalium or its products can also inhibit the activation and proliferation of T lymphocytes (31). However, the specific mechanism and possible pathway through which M. genitalium inhibits T lymphocyte activation requires further study.

The CaN-NFAT signaling pathway plays a crucial role in promoting the expression of cytokines, such as IL-2 and IFN-γ, leading to the activation of T cells (32). Downregulation of CaN phosphorylation inhibits NFAT from entering the nucleus, inhibiting T cells activation. Therefore, CaN inhibitors, including as CsA, FK506, and Pimecrolimus, are frequently utilized in clinical settings as an immunosuppressive therapeutics (33). Pimecrolimus was chosen as the CaN inhibitor in this investigation, and CaN and MgPa were used to pretreat T cells, respectively. PMA/ionomycin was used to induce T cell activation and detect CaN phosphorylation and nuclear translocation of NFAT. Similar to CaN inhibitors, MgPa inhibited PMA/ionomycin-induced CaN phosphorylation and attenuated the translocation of NFAT into the nucleus in Jurkat cells. The results were similar to those of H. pylori VacA inhibiting T cell activation by regulating the CaN-NFAT signaling pathway (34–36). Furthermore, the interference of CypA with siRNA and CypA-deficient mice weakened the ability of MgPa to inhibit CaN phosphorylation in T cells, indicating that the interaction between MgPa and CypA could negatively regulate T cell activation through the CaN-NFAT signaling pathway.

CD69, the surface marker in the early stage of T cell activation, can be expressed on the membrane surface of mature T cells for a long time and acts as a co-stimulatory signal to promote T cell activation and proliferation (37). CD25 is the signal of T cell metaphase activation (38–40). The findings of this investigation revealed that rMgPa could downregulate the expressions of CD69 and CD25 and, thus, inhibit CD3/CD28 MAb-induced T cells activation. This result is similar to that of the Shigella flexneri secretion system, which inhibited T cell activation by downregulating the CD69 and CD25 expression (30). The CD69 and CD25 expressions increased in T cells that CypA was interfered by CypA-siRNA or CypA-knockout mice, which indicated that M. genitalium could inhibit the expressions of CD69 and CD25, the molecules related to T cell activation, through MgPa-CypA interaction.

The immune system defense against mycoplasma infections depends heavily on cytokines. IL-2 is an important cytokine involved in T cells activation. The binding of IL-2 and the corresponding IL-2 receptor on the T cell membrane surface can induce the activation of interleukin-2 inducible T cell kinase (ITK). ITK, a member of the tyrosine kinase Tec family, can act as a downstream molecule of the TCR signaling pathway and promote T cell proliferation (31, 41). IFN-γ was produced by Th1 and natural killer (NK) cells and acts as an activator of mononuclear macrophages by increasing antigen presentation and promoting T cell aggregation at lesion sites (42) which is an indicator of T cells activation. Our findings demonstrated that cytokines associated with T cells activation, such as IL-2 and INF-γ, as well as the surface markers CD25 and CD69, were considerably reduced in rMgPa-stimulated T cells, demonstrating that rMgPa might reduce CD3/CD28 MAb or PMA/ionomycin-induced T cells activation. The results were different from those of HIV FP fragment/virus-polysaccharides Vi, which inhibited CD3/CD28 MAb-mediated T cell activation but did not inhibit PMA/ionomycin-induced T cells activation, possibly because rMgPa inhibited T cell activation by subduing CaN phosphorylation in the downstream of TCR pathway other than the TCR receptor (43).

Jurkat cells are a better model for T cell activation. However, their cell activation effect is not as good as that of human primary T cells. Therefore, PBMCs were extracted from the blood of healthy volunteers to further verify the inhibitory effect of the MgPa-CypA interaction on IFN-γ, IL-2, CD69, and CD25 expressions. Our results suggested that rMgPa can downregulate the expressions of these T cell activation-related molecules. However, the B cells contained in PBMCs could also induce the expression of cytokines and CD molecules. To avoid the interference from B cells, we verified that rMgPa could also subdue the expressions of these molecules in the magnetic bead method-purified T cells. Because the amino acid homology between mouse CypA and human CypA is high (up to 95.67%), we investigated the effects of rMgPa on the expression of T cell activation-related molecules in normal mice and CypA-knockout mice T cells. In conclusion, our results clearly demonstrated that rMgPa could suppress T cell activation by downregulating the CypA-CaN-NFAT pathway, and therefore CypA may be a potential new therapeutic target for M. genitalium infection.

## MATERIALS AND METHODS

### Cell source and culture.

Jurkat cells were obtained from Chinese Academy of Sciences Shanghai Cell Bank and cultured in Roswell Park Memorial Institute (RPMI)-1640 medium with 10% FBS incubated in a 5% CO2 incubator at 37°C. Peripheral blood mononuclear cells (PBMCs) were isolated from venous blood collected from healthy volunteers by Ficoll-Paque centrifugation. Additionally, the anti-human CD3 Magnetic Particles-DM (BD Biosciences) was used to isolate the purified CD3^+^ T cells from PBMCs using positive magnetic bead selection.

CypA-knockout mice were constructed and identified by Cyagen (Suzhou) Biotechnology Co, Ltd. (). Under aseptic circumstances, spleens from CypA-knockout mice and healthy C57BL/6 mice were harvested. Cell numbers were counted with a hemocytometer (LO-Laboroptik Ltd.) using trypan blue staining. Purified CD3^+^ T cells were obtained from the spleens of C57BL/6 mice with negative magnetic bead selection according to the operating instruction of the Mouse T Lymphocyte Enrichment Set-DM (BD Biosciences) by Flow Cytometry.

### Location of CypA on Jurkat cells.

Jurkat cells were fixed for 15 to 30 min with 4% paraformaldehyde at 4°C, incubated for 1 h with FITC-conjugated goat anti-mouse IgG (1:100 Beyotime), and then treated for 1 h with mouse anti-CypA antibody (1:100, Abcam), and 4,6-diamidine-2-phenylindole dihydrochloride (DAPI) was added to visualize nuclear DNA. An inverted microscope was used to examine the distribution of CypA after 3 PBS washes. (Nikon, TE2000-S).

### Co-localization analysis between rMgPa and CypA in Jurkat cells.

The expression and purification of recombinant MgPa (rMgPa, 1,075 to 1,444 aa) and the preparation of rabbit anti-rMgPa antibody were performed as described in our previous study (44). In addition, the co-localization of between rMgPa and CypA in Jurkat cells was analyzed using indirect immunofluorescence. After being washed with PBS, Jurkat cells were stimulated with rMgPa for 2 h at 37°C, and incubated with rabbit anti-rMgPa (1:2,000) and mouse anti-CypA (1:200, Abcam) antibodies for 2 h at 37°C, and Jurkat cells were used. Following washing, cells were incubated for 1 h at 37°C with FITC- and Cy3-labeled sheep anti-mouse and sheep anti-rabbit IgG antibodies from Proteintech and Beyotime, respectively. Co-localization was examined under an inverted microscope, following washing.

### Detection of the CaN phosphorylation in Jurkat cells.

Cells were pre-stimulated with pimecrolimus (30 μmol/L), a CaN inhibitor, or rMgPa (30 μg/mL) for 30 min and then incubated with Phorbol 12-Myristate 13-Acetate (PMA)/ionomycin (500 ng/mL PMA and 10 μg/mL Ionomycin). Simultaneously, cells were transfected with CypA-siRNA (Origene Technologies) using Lipofectamine 3,000 according to the manufacturer's instructions. Cells were then harvested and solubilized for 1.5 h at 4°C in lysis buffer containing a protease inhibitor mixture. CaN was isolated using SDS-PAGE and then transferred to a polyvinylidene fluoride membrane. The primary and secondary antibodies were designated as the phosphorylated CaN antibody (1:200, Abcam) and the HRP-conjugated goat anti-rabbit IgG antibody (Proteintech), respectively. CaN phosphorylation was detected by WB using a chemiluminescent substrate using ImageJ software.

### Indirect immunofluorescence detection of NFAT.

Indirect immunofluorescence assay (IFA) was performed to investigate whether rMgPa can inhibit the nuclear translocation of NFAT. Jurkat cells stimulated with CaN inhibitor (Pimecrolimus, 30 μmol/L), rMgPa (30 μg/mL) or transfected with CypA-siRNA, respectively, in the presence of PMA/ionomycin (500 ng/mL PMA and 10 μg/mL Ionomycin) were set up for the experimental group. Jurkat cells activated with PMA/ionomycin were used positive control, and PBS-stimulated cells were used negative control. NFAT was probed with rabbit anti-NFAT antibody (CST) overnight at 4°C, followed by incubation with Cy3-conjugated AffiniPure goat anti-rabbit IgG (H+L) antibody for 1 h at room temperature. After being washed with PBS, all samples were analyzed using an inverted microscope (ZEISS).

### Real-time quantitative PCR.

The mRNA levels of IL-2, IFN-γ were measured in Jurkat cells using Real-time quantitative PCR (RT-qPCR). The experimental grouping and cell treatment factors were the same as the detection of nuclear translocation of NFAT by IFA. TRIzol (Beyotime) was used to extract the total RNA from T cells, and the Beyo RT II First Strand cDNA synthesis kit was used to reverse-transcribe the RNA into cDNA (RNase H minus). RT-qPCR was carried out with a Light Cycle 96 device (Roche, Basel). The specific primer sequences were designed as follows: IL-2 (human) forward: 5′-CACCAGGATGCTCACATTTAAG-3′ and reverse: 5′-CTCCAGAGGTTTGAGTTCTTCT-3′; INF-γ (human) forward: 5′-GAGATGACTTCGAAAAGCTGAC-3′ and reverse: 5′- CCTTTTTCGCTTCCCT GTTTTA-3′ and GAPDH forward: 5′-GCACCGTCAAGGCTGAGAAC-3′ and reverse: 5′-TGG GAAGACGCCAGTGGA-3′. All primers were Purchased from Shanghai Sangong, Shanghai, China, Biotechnology Co. Ltd. The PCR settings were 95°C for 10 min, 95°C for 15 s, 72°C for 20 s, and 56°C for 15 s. The expression levels of glyceraldehyde 3-phosphate dehydrogenase (GAPDH) in each sample were normalized to the data.

### Cytokine quantification by ELISA.

The expressions levels of IL-2 and IFN-γ were determinated in Jurkat cells using indirect ELISA. Jurkat cells were pre-stimulated 30 min with Pimecrolimus (30 μmol/L), rMgPa (30 μg/mL) or transfected with CypA-siRNA, respectively, and then incubated with CD3/CD28 MAb (20 ng/mL, IBA) for 48 h. Jurkat cells activated with CD3/CD28 MAb were used positive control, and PBS-stimulated cells were used negative control. The IFN-γ and IL-2 in the supernatants were analyzed using ELISA kits (Thermo Fisher Scientific) according to the manufacturer's instructions.

### Analysis of IL-2, IFN-γ, CD69, and CD25 by FCM.

Flow cytometry (FCM) was used to detect the expressions of IL-2, IFN-γ, CD69, and CD25 in order to determine whether rMgPa can limit T cells activation, PBMCs or purified T cells were activated with 20 ng/mL of CD3/CD28 MAb stimulation and CypA-siRNA, CaN inhibitor (30 mol/L) pre-incubation, and rMgPa (30 g/mL) pre-incubation groups were established. Cells were stained with PE-conjugated mouse anti-human CD69 MAb (BD), PerCP-Cy5.5-conjugated mouse anti-human CD25 MAb (BD), PerCP-Cy5.5-anti-Mouse CD69 (BD), and FITC-anti-mouse CD25 (BD). After that, the cells were fixed, permeabilized, and stained with APC-anti-human IL-2 (BD), FITC-anti-human IFN- (BD), APC-anti-mouse IL-2 (BD), and PE-anti-mouse IFN (BD), respectively. Finally, the cells were resuspended with PBS containing 0.5% BSA, IFN-γ, IL-2, CD25, and CD69 expression were analyzed by Fluorescence-activated cell sorting using Cell Quest Pro software.

### Statistical analysis.

Values were calculated as the mean ± SD and statistical analysis was conducted using an unpaired Student's *t* test. *, *P < *0.05 were considered statistically significant in all analyses.

### Conclusions.

In summary, this study confirmed that rMgPa could interact with CypA, downregulating CaN phosphorylation and inhibiting NFAT nucleation and the expression of surface-activated molecules of T cells, such as CD69 and CD25. In addition, it downregulated intracellular cytokines IL-2 and INF-γ, demonstrating that rMgPa plays an immunosuppressive role in T cells via the CypA-CaN-NFAT pathway. Our findings confirmed that M. genitalium could interact with host T cells by MgPa-CyPa interaction and clarified the immunosuppression mechanism of M. genitalium to host T cells.

### Ethics approval.

All experiments were approved by Human Subject Research and the Ethics Committee on Animal Experiments of University of South China (permit number: 4304079008946) on August 18th, 2021. All human research was performed according to the Declaration of Helsinki and all animal research in accordance with the Basel Declaration.

### Availability of data and materials.

The data sets generated for this study are available on request to the corresponding author.
